# Is there an association between mean platelet volume and diabetic retinopathy? A case-control study

**DOI:** 10.22088/cjim.12.2.129

**Published:** 2021-03

**Authors:** Seyed Ahmad Rasoulinejad

**Affiliations:** 1 Clinical Research Development Unit of Rohani Hospital, Babol University of Medical Sciences, Babol, Iran; 2 Department of Ophthalmology, School of Medicine, Babol University of Medical Sciences, Babol, Iran

**Keywords:** Retinopathy, Diabetes mellitus, Mean platelet volume

## Abstract

**Background:**

To the aim of this study was to evaluate the association between mean platelet volume (MPV) and diabetic retinopathy (DR).

**Methods:**

Patients with diabetes mellitus (DM) referred to Ophthalmology Clinic of Rohani Teaching Hospital in Babol, Northern Iran were entered into this case-control study. Healthy subjects in control group included individuals without history of DM. The patients were classified into four groups including I. Control (n=79), II. Diabetic patients without DR (n=68), III. Non-proliferative DR (n=61), and IV. Proliferative DR (n=64). Blood samples were collected, and necessary laboratory tests were performed.

**Results:**

The MPV value was significantly higher in each group of II, III and IV compared to group I (p<0.001). This value was also significantly higher in each group of III and IV compared to group II. On the other hand, there was no significant difference between III and IV groups in MPV. A significant correlation was found between MPV and fasting blood sugar in groups II (r=0.349, p=0.004), III (r=0.269, p=0.036) and IV (r=0.258, p=0.040). Moreover, there was a significant correlation between MPV and hemoglobin A1c in groups II (r=0.366, p=0.002), III (r=0.312, p=0.015) and IV (r=0.278, p=0.026).

**Conclusion:**

The results of this study showed that the increased MPV value was directly associated with DR and its severity. A positive association was also found between MPV and indicators of glycemic status. Considering that measurement of MPV as a suitable parameter reflecting platelet function can be easily conducted, it can be clinically used to monitor status of DR.

Diabetes mellitus (DM) is a chronic metabolic disorder associated with chronic hyperglycemia, and long-term macro- and microvascular consequences involving eyes, kidneys and nerves, generally associated with oxidative stress. The majority of morbidity and mortality associated with DM are due to the hyperglycemia and its macro-and microvascular complications (1-3). Measuring fasting blood glucose, postprandial blood glucose and glycated hemoglobin are essential for monitoring glycemic control of DM patients. Hemoglobin A1c (HbA1c) is extensively applied as a marker of chronic glycemia, reflecting the mean blood glucose levels for 2-3 months (4, 5). Diabetic retinopathy (DR) is frequently observed in patients with DM, which is the main causative agent of loss of vision. The factors affecting DR include length of DM, genetics, smoking, abdominal obesity, hypertension, hyperglycemia and urinary albumin (6-8).

Elevated mean platelet volume (MPV) has been a marker for platelet activation. The DM has been associated with platelet hyperreactivity, hyperaggregability, increased thrombogenesis and decreased fibrinolysis regardless diabetes type (9, 10). Thrombogenesis and atherogenesis are related to platelet activation and activity. Increased MPV values result in arterial thrombotic events like myocardial infarction as well as cerebral thromboembolism (11, 12). Studies reported that the MPV can be increased in diabetic patients (11, 13). The role of large platelets may be related to the vascular injury growth in DM by increased activation, producing more prothrombotic factors or endothelium-associated vasomotor dysfunction. However, the role of MPV to develop diabetic vascular disorder has not yet been identified (14, 15). A limited number of studies have been conducted on the relationship between DM and platelet parameters; therefore, the aim of this study was to investigate this association. 

## Methods

**Study population: **In this case-control study, the patients with DM referred to the Ophthalmology Clinic of Rohani Teaching Hospital in Babol, Northern Iran, were recruited during routine outpatient visits from April 2018 to August 2019. Diabetic patients were diagnosed according to the World Health Organization diagnostic criteria (16).

The exclusion criteria included type 1 diabetes, suspicion of pregnancy, glomerular filtration rate of smaller than 60 mg/dL, cardiac disease and history of diseases due to the impaired thyroid function, active infections and anemia. Cases with inherited or acquired platelet disorders, hematologic diseases and acute stress as well as cases who had received anticoagulant and/or antiplatelet therapies (due to affecting MPV) were excluded from the present study. In addition, the smokers were excluded since the MPV levels may be influenced by the smoking (17). The healthy subjects in the control group included individuals without DM history. 

**Sample size determination: **Fifty cases were in each group considering clinical judgment with 80% power and type I error of 5%. The mean MPV for DR and control groups was 9.5 and 9, respectively with standard deviation of 1 for both groups. Sampling ratio was 1:1. 

**Clinical evaluation:** All clinical examinations were performed by one expert ophthalmologist. The DR was regarded as a minimum of one microaneurysm, retinal hemorrhage and/or other symptoms of retinal injury (18).

**Blood sampling and laboratory tests:** Blood sample from each subject was collected after the clinical evaluation of DR. Sampling was done in fasted state in the morning for preventing the influence of daily variation on the hemostatic system in the laboratory of Ayatollah Rohani Hospital. Blood sampling was done with minimum stasis in EDTA Vacutainer Tubes and evaluated in 60 min. Fasting blood sugar (FBS), HbA1c, white blood cell (WBC) count, Hb, platelet count and MPV were measured. The MPV reference range of 7.4–10.4 fL was regarded.

**Statistical analysis:** Data were analyzed using SPSS. Normality of the data was tested using the Kolmogorov-Smirnov test. For comparing the parametric data between the groups, Student’s t-test (between any two groups) and ANOVA test (between the four study groups) were applied, and for comparison of non-parametric data between the groups, the Kruskal–Wallis test was used. Analysis of covariance (ANCOVA) was applied for comparison of data between the groups adjusted for covariates of FBS and HbA1c. To assess any correlations between the quantitative variables, the Pearson correlation test and Spearman's rank test were utilized when parametric and non-parametric conditions existed, respectively. Chi-square test was used for gender variable. A p-value of <0.05 was considered to be significant in all tests.

**Ethical issues:** The Research Ethical Committee of Babol University of Medical Sciences and Health Services approved the study (code: IR.MUBABOL.HRI.REC.1398.146). All individuals provided written informed consent.

## Results

Out of 272 patients, 148 (54.4%) and 124 (45.6%) were men and women, respectively. The subjects’ average age was 54.33±9.76, ranging from 31 to 76 years old. A number of 79, 68, 61 and 64 subjects were enrolled in the groups of control (group I), diabetic patients without DR (group II), non-proliferative DR (NPDR, group III) and proliferative DR (PDR, group IV), respectively. The number of men in groups I, II, III and IV was 47 (59.5%), 38 (55.9%), 33 (54.1%) and 30 (46.9%), which did not show a significant difference. Comparing the mean values of the qualitative variables between the groups including FBS, HbA1c, WBC, Hb and platelet count have been represented in table 1. Table 2 shows comparison of the platelet count and MPV between the study groups. As indicated, according to the ANOVA test, there was a significant difference between the groups in MPV value (p<0.001). In addition, Student’s t-test indicated that the MPV value was significantly higher in the II, III and IV groups than group I (p<0.001). 

This value was also significantly higher in each group of III and IV than group II. In contrast, groups III and IV demonstrated no significant differences in MPV. Additionally, no significant difference was identified between four groups in platelet count based on ANOVA test. After adjustment for FBS and HbA1c, the ANCOVA test also illustrated significant differences between groups in MPV (p<0.001). Overall, there was not a significant correlation between MPV and platelet count according to the Pearson correlation test. Similar lack of significance was observed for the four study groups separately. Overall, the MPV and FBS were significantly correlated with each other (r=0.442, p<0.001) according to the Spearman's rank test. Similar significant correlation was seen in groups II (r=0.349, p=0.004), III (r=0.269, p=0.036) and IV (r=0.258, p=0.040). Spearman's rank test showed that there was a significant correlation between MPV and HbA1c (r=0.464, p<0.001) in whole subjects. Similar significant correlation was observed in groups II (r=0.366, p=0.002), III (r=0.312, p=0.015) and IV (r=0.278, p=0.026).

**Table 1:** Comparison of qualitative variables between four study groups

**Variables**	**Group I (n=79)**	**Group II (n=68)**	**Group III (n=61)**	**Group IV (n=64)**	**P-value**
	**Mean±SD**	**Mean±SD**	**Mean±SD**	**Mean±SD**	
Age (years)	53.44±10.04	55.78±8.86	55.87±9.21	52.52±10.59	0.112^a^
FBS* (mg/dL)	89.38±12.09	197.91±81.61	209.11±78.23	231.20±77.21	<0.001^b^
HbA1c** (%)	5.96±0.38	9.44±2.76	9.83±2.64	10.32±2.34	<0.001^b^
WBC*** (µL)	8043.04±1494.24	7829.41±1505.32	7772.13±1697.86	8231.25±1661.98	0.337^a^
Hb (g/dL)	13.22±0.82	13.45±1.08	13.49±1.04	13.16±1.03	0.141^a^

Group I, healthy controls without diabetes; Group II, diabetic patients without retinopathy; Group III, non-proliferative diabetic retinopathy; Group IV, proliferative diabetic retinopathy

* Fasting Blood Sugar ** Hemoglobin A1c

*** White Blood Cell

**** MPV: Mean platelet volume

a, based on Analysis of Variance (ANOVA) test; b, based on Kruskal-Wallis Test

**Table 2:** Comparison of platelet count and mean platelet volume values between four study groups

**Variables**	**Group I (n=79)**	**Group II (n=68)**	**Group III (n=61)**	**Group IV (n=64)**	**P-value**
	**Mean±SD**	**Mean±SD**	**Mean±SD**	**Mean±SD**	
Platelet count	287.23±52.09	290.62±53.27	297.13±57.96	295.98±51.76	0.667^a^
Mean platelet volume (fL)	8.74±1.03	9.09±0.98	9.67±0.93	9.84±0.96	<0.001^a^

Group I, healthy controls without diabetes; Group II, diabetic patients without retinopathy; Group III, non-proliferative diabetic retinopathy; Group IV, proliferative diabetic retinopathy a, based on Analysis of Variance (ANOVA) test

## Discussion

The association between MPV and DR was investigated. As shown, diabetics with PDR and NPDR had significantly higher MPV compared with the normal group. The increased MPV in DM cases with PDR and NPDR was similar to other studies (19, 20). A recent meta-analysis including 14 articles conducted by Ji et al. (11)indicated that MPV values were significantly more in DR group in comparison with healthy controls and in diabetic group without retinopathy. Furthermore, they found that the MPV level was higher in PDR group compared to diabetic group without retinopathy. Additionally, no significant difference was found between PDR and non-PDR groups in MPV (11). Other meta-analysis by Ji et al. (21) also suggested a significant elevation in platelet distribution width level in DR group and control group versus diabetic patients without DR. Regarding platelet count, the analyses showed a reduction in DR cases than DM ones with no DR and no differences in DR group compared to control group.

Disturbances in DM and related consequences are associated with alterations in platelet performance and shape. Platelets are effective in capillary nonperfusion in DM. Studies also have reported that the platelet is crucial to develop DR, but not necessarily initiates the pathology of early DR (11, 22, 23). Some theories have been mentioned to describe the relation between platelet and DR. For example, thrombogenesis and microvascular lesions by platelet have been noted as a mechanism (24). In between, factors releasing by platelet like platelet-derived growth factor are efficient (25).

High MPV indicates large platelet size as well as more platelet adhesion and aggregation. Large platelets have found more active, containing denser granules, which generate higher amount of β-thromboglobulin, serotonin and thromboxane A2, potentially associated with higher risk of vascular complications (26, 27). Larger MPV is associated with more likelihood of thrombosis formation whereas vascular endothelial damage can trigger platelet adhesion and aggregation for accelerated thrombosis (28). There are no enough data on the main action of elevated MPV in DM. It is believed that osmotic swelling of the platelets occurs because of high blood glucose or glucose metabolites (29). Exogenous or endogenous insulin is effective because it makes megakaryocytes producing bigger platelets (30). Shorter life spans of platelets in DM as well as young platelets that are large are also effective in this regard (31, 32). Despite of shorter life of platelets in DM (33), the platelet size is not correlated to age and is measured when it is synthesized from the megakaryocyte (34).

In the ongoing study, a mild and positive correlation was observed between MPV and indicators of glycemic status (HbA1c and FBS) in diabetic patients. Limited studies exist investigating the mentioned association, especially among general population, and inconsistent results have been reported. Some studies found positive correlations between MPV and glycemic status among general population (35-37) while other results showed a similar correlation only in diabetic patients (38). Therefore, more studies are necessary to be performed to make this association clear. If there is such association in general population (and not only among diabetic patients), detection of people with activated platelets to intervene to prevent future thrombotic events will be conducted more easily and without additional costs.

In the present study, it was observed that increased MPV value was directly associated with DR and its severity. Moreover, a positive relationship was found between MPV and indicators of glycemic status. Considering that measurement of MPV as a suitable parameter reflecting platelet function can be easily conducted, it can be clinically used to monitor status of DR. 
